# Prognostic factors in pulmonary metastasectomy and efficacy of repeat pulmonary metastasectomy from colorectal cancer

**DOI:** 10.1186/s12957-020-02076-3

**Published:** 2020-11-30

**Authors:** Masahiro Fukada, Nobuhisa Matsuhashi, Takao Takahashi, Yoshihiro Tanaka, Naoki Okumura, Hirotaka Yamamoto, Koyo Shirahashi, Hisashi Iwata, Kiyoshi Doi, Kazuhiro Yoshida

**Affiliations:** 1 Department of Surgical Oncology, Graduate School of Medicine, Gifu University, 1-1 Yanagido, Gifu City, Gifu, 501-1194 Japan; 2 Department of General and Cardiothoracic Surgery, Graduate School of Medicine, Gifu University, 1-1 Yanagido, Gifu City, Gifu, 501-1194 Japan

**Keywords:** Colorectal cancer, Pulmonary metastasis, Repeat pulmonary metastasectomy, Prognostic factor, Mediastinal lymph node metastasis

## Abstract

**Background:**

The rate of pulmonary metastasectomy from colorectal cancer (CRC) has increased with recent advances in chemotherapy, diagnostic techniques, and surgical procedures. The purpose of this study was to investigate the prognostic factors for response to pulmonary metastasectomy and the efficacy of repeat pulmonary metastasectomy.

**Methods:**

This study was a retrospective, single-institution study of 126 CRC patients who underwent pulmonary metastasectomy between 2000 and 2019 at the Gifu University Hospital.

**Results:**

The 3- and 5-year survival rates were 84.9% and 60.8%, respectively. Among the 126 patients, 26 (20.6%) underwent a second pulmonary metastasectomy for pulmonary recurrence after initial pulmonary metastasectomy. Univariate analysis of survival identified seven significant factors: (1) gender (*p* = 0.04), (2) past history of extra-thoracic metastasis (*p* = 0.04), (3) maximum tumor size (*p* = 0.002), (4) mediastinal lymph node metastasis (*p* = 0.02), (5) preoperative carcinoembryonic antigen (CEA) level (*p* = 0.01), (6) preoperative carbohydrate antigen 19-9 (CA19-9) level (*p* = 0.03), and (7) repeat pulmonary metastasectomy for pulmonary recurrence (*p* < 0.001). On multivariate analysis, only mediastinal lymph node metastasis (*p* = 0.02, risk ratio 8.206, 95% confidence interval (CI) 1.566–34.962) and repeat pulmonary metastasectomy for pulmonary recurrence (*p* < 0.001, risk ratio 0.054, 95% CI 0.010–0.202) were significant. Furthermore, in the evaluation of surgical outcomes, the safety of second pulmonary metastasectomy was almost the same as that of initial pulmonary metastasectomy.

**Conclusions:**

Repeat pulmonary metastasectomy is likely to be safe and effective for recurrent cases that meet the surgical criteria. However, mediastinal lymph node metastasis was a significant independent prognostic factor for worse overall survival.

## Introduction

Colorectal cancer (CRC) is one of the most common cancers and is known to metastasize frequently to the liver and lungs via the systemic blood flow. In the past, pulmonary metastasis was considered to be an indicator of cancer spread throughout the body, and aggressive treatment was commonly avoided. However, because of recent advances in chemotherapy, diagnostic techniques, and surgical procedures, pulmonary resection is widely accepted as the optimal treatment for pulmonary metastases [1–8]. It is expected that the clinical results of pulmonary metastasectomy will change with the improvement of medical care. Thus, it is necessary to continue evaluation of the outcome of pulmonary metastasectomy in CRC patients in order to identify true prognostic factors and determine appropriate surgical criteria. We report the recent clinical outcomes of pulmonary metastasectomy at our institutes. The main purpose of this study was to answer the following questions: (1) What are the potential prognostic factors for patients undergoing pulmonary metastasectomy? (2) What is the role of repeat pulmonary metastasectomy for recurrent metastatic CRC?

## Patients and methods

### Study population

**Fig. 1:**
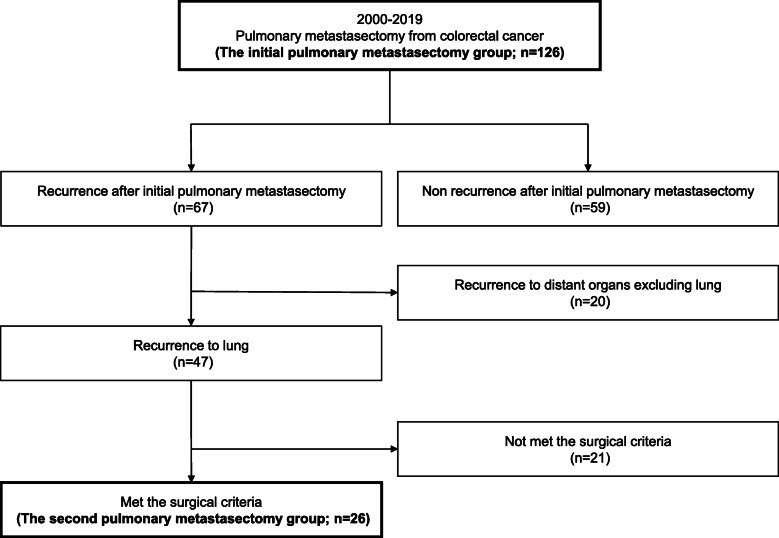
Patients’ flow diagram of this study

A total of 126 patients underwent pulmonary metastasectomy at the Department of Thoracic Surgery, Gifu University Hospital, between March 2000 and December 2019. The study’s retrospective protocol was approved by our institutional review board (approval number ‘2019-253’). Among the 126 patients included, 47 (37.3%) had pulmonary recurrence after initial pulmonary metastasectomy, and 26 (20.6%) who met the surgical criteria underwent a second pulmonary metastasectomy (Fig. 1).

All patients who underwent pulmonary metastasectomy met the following criteria based on the Japanese Society for Cancer of the Colon and Rectum (JSCCR) Guidelines for the treatment of CRC [9]: (1) the patient was capable of tolerating surgery; (2) the primary colorectal tumor was controlled or could be controlled; (3) the metastatic lung tumor could be completely resected; (4) any extra-thoracic metastases could be controlled; and (5) the function of the remaining lung would be adequate.

A controllable tumor is a tumor that can be completely resected, or a tumor without the appearance of new lesions and regrowth after treatment such as surgery, chemotherapy, and radiation.

Preoperative assessments included clinical examination, blood tests, electrocardiogram, standard chest radiograph, spirometry, echocardiogram, contrast-enhanced computed tomography scan (CT) of the chest and abdomen, and positron emission tomography (PET) whole body scan. Endobronchial ultrasound-guided transbronchial biopsy was not performed routinely in this study.

Regarding the extent of pulmonary resection, if metastatic tumor was anatomically present in the outer third of the lung and partial resection was possible, we performed partial resection. If it was located inside the lung, segmentectomy and lobectomy were performed.

Mediastinal lymph node dissection was performed according to primary lung cancer in the cases in which lymph node metastasis was suspected (swelling with short axis on CT ≧ 10 mm and PET-positive). If lymph node metastasis was suspected during surgery, this lymph node was submitted to intraoperative consultation. If positive, mediastinal lymph node dissection was performed. In addition, in the case of lobectomy and segmentectomy, the regional and interlobar lymph nodes were dissected. Otherwise, mediastinal lymph node dissection was not performed.

The surgery was considered curative if all known pulmonary nodules were removed. Patients who had complete resection of all known pulmonary disease were included in this study. We reviewed each patient’s medical records to obtain clinicopathological information of the initial and second pulmonary metastasectomy.

We collected information on patients and primary colorectal tumor characteristics including gender, age at the initial pulmonary metastasectomy, smoking habits (non-smoker or smoker), Brinkman index, primary colorectal tumor location (colon or rectum/right or left side), histological differentiation of the primary colorectal tumor (well, moderately, or poorly differentiated), pathological Union for International Cancer Control-TNM classification (8th edition) [10] of the primary colorectal tumor, past history of extra-thoracic metastasis (present or absent), adjuvant chemotherapy after the primary colorectal operation (yes or no), and the number of pulmonary metastasectomies.

The clinical characteristics of pulmonary metastases included diagnosis period (synchronous or metachronous), number (solitary or multiple), location (unilateral or bilateral), disease-free interval, maximum tumor size, mediastinal lymph node metastasis (positive or negative in postoperative histological lymph node status), preoperative carcinoembryonic antigen (CEA) (normal or elevated, normal upper limit being 5 ng/ml), preoperative carbohydrate antigen 19-9 (CA19-9) (normal or elevated, normal upper limit being 37 ng/ml), perioperative chemotherapy (yes or no), recurrence after pulmonary metastasectomy (yes or no), and recurrent distant organ. In this study, lung lesions diagnosed within 1 year from resection of the primary colorectal tumor were defined as synchronous metastases, and those diagnosed after 1 year were defined as metachronous metastases. The disease-free interval (DFI) referred to both the period from primary colorectal tumor resection to diagnosis of the initial pulmonary metastasis and the period from the initial pulmonary metastasectomy to diagnosis of the second pulmonary metastasis. In our department, as a general rule, perioperative chemotherapy was indicated in cases excluding solitary pulmonary metastasis with DFI > 1 year.

Finally, the surgical characteristics of pulmonary metastasectomy were operation method (partial resection, segmentectomy, lobectomy/video-assisted thoracic surgery (VATS), or open surgery), operation time, intraoperative blood loss, preoperative percent vital capacity (%VC), preoperative forced expiratory volume percent in 1 s (FEV_1.0_%), preoperative respiratory dysfunction (absent or present), postoperative complications after pulmonary metastasectomy (Clavien-Dindo classification [11] grade ≥ 2: yes or no), postoperative mortality, and hospital stay.

### Statistical analysis

For comparisons of variables between the initial and second pulmonary metastasectomy groups, Fisher’s exact test was used for categorical variables, and the Mann-Whitney *U* test was used for continuous and ordinal variables.

Overall survival was calculated in months from the date of the initial pulmonary resection to the date of the last follow-up. All cumulative survival curves were estimated using the Kaplan-Meier method, and in the univariate analysis, the log-rank test was used to evaluate differences between groups. A Cox relative risk regression model was used to estimate risk ratios and 95% confidence intervals (CIs) for multivariate analysis. The significance level was set at < 0.05. All statistical analyses were performed using JMP software (SAS Institute Inc., Cary, NC, USA).

## Results

### Patient demographics

Patient and primary colorectal tumor characteristics for each group are presented in Table 1. The cohort consisted of 85 males (67.5%) and 41 females (32.5%). The age at the initial pulmonary metastasectomy ranged from 37 to 84 years, with a median of 66 years. The primary tumor location was the colon in 59 cases (46.8%) and the rectum in 66 cases (52.4%). Forty-five patients (35.7%) had a past history of extra-thoracic metastasis, and the liver (33 cases, 26.2%) was the most frequent site of metastasis.

**Table 1:** Patients and primary colorectal tumor characteristics in each group (initial and second pulmonary metastasectomy)

Characteristics	The initial pulmonary metastasectomy *n* = 126	The second pulmonary metastasectomy *n* = 26	*p* value
Gender, *n* (%)	Male 85 (67.5) Female 41 (32.5)	Male 16 (61.5) Female 10 (38.5)	0.56
Age¶, median [range]	66 [37–84]	62.5 [37–77]	0.04^*^
Smoking habits, *n* (%)	Non-smoker 39 (31.0) Smoker 79 (62.7)	Non-smoker 10 (38.5) Smoker 13 (50.0)	0.34
Brinkman index✝, median [range]	457.5 [0–2100]	107 [0–2000]	0.46
Primary colorectal tumor location, *n* (%)	Colon 59 (46.8) Rectum 66 (52.4)	Colon 11 (42.3) Rectum 16 (57.7)	0.65
	Right side 23 (18.2) Left side 102 (81.0)	Right side 3 (11.5) Left side 23 (88.5)	0.38
Histological differentiation of the primary colorectal tumor, *n* (%)	Well–55 (43.6) Moderately–53 (42.0) Poorly–6 (4.8)	Well–8 (30.8) Moderately–9 (34.6) Poorly–1 (3.8)	0.54
Pathological T stage‡, *n* (%)	T1 7 (5.6) T2 11 (8.7) T3 61 (48.4) T4 37 (29.4)	T1 1 (3.8) T2 0 (0.0) T3 15 (57.7) T4 6 (23.1)	0.19
Pathological N stage‡, *n* (%)	N0 43 (34.1) N1 47 (37.3) N2 24 (19.0)	N0 7 (27.0) N1 9 (34.6) N2 5 (19.2)	0.92
Pathological stage‡, *n* (%)	I 8 (6.3) II 25 (19.8) III 56 (44.4) IV 31 (24.6)	I 0 (0.0) II 5 (19.2) III 12 (46.2) IV 8 (30.8)	0.35
Past history of extra thoracic metastasis, *n* (%)	Present 45 (35.7) Absent 81 (64.3)	Present 10 (38.5) Absent 16 (61.5)	0.79
Adjuvant chemotherapy, *n* (%)	Yes 61 (48.4) No 57 (45.2)	Yes 16 (61.5) No 8 (30.8)	0.18
Regimen, *n* (%)	UFT 28 (22.2) capecitabine 7 (5.6) CapeOX 7 (5.6) FOLFOX 5 (4.0) S1 4 (3.2) FU + LV 3 (2.4) Other 7 (5.6)	UFT 5 (19.2) FOLFOX 3 (11.5) Capecitabine 2 (7.7) CapeOX 1 (3.8) FU + LV 1 (3.8) Other 4 (15.4)	–
Number of pulmonary metastasectomy, *n* (%)	–	2 times 14 (53.9) 3 times 7 (26.9) 4 times 5 (19.2)	–

¶: Age at the initial pulmonary metastasectomy

✝: Brinkman index = (the number of cigarette smoked per day) × (the number of years of smoking)

‡: UICC TNM classification(the 8^th^ edition)

^*^p < 0.05; ^**^p < 0.01; ^***^p < 0.001

In the second pulmonary metastasectomy group, the mode and maximum number of repeat pulmonary metastasectomies were 2 (14 cases, 53.9%) and 4 (5 cases, 19.2%), respectively. The age at the initial metastasectomy was significantly younger (*p* = 0.04). However, there was no significant difference in other patient and primary colorectal tumor characteristics between the two groups.

The characteristics of pulmonary metastases in each group are presented in Table 2. There was no significant difference between the two groups in 10 clinical characteristics. Although the DFI was not significantly different between the groups {median 541.5 (range 0–4664) days vs 409 (range 27–1334) days, *p* = 0.13}, the recurrence rate tended to be higher in the second pulmonary resection group (53.2% vs 65.4%, *p* = 0.07). The lung was the most common metastatic organ in both groups (37.3% and 46.4%, respectively).

**Table 2:** Pulmonary metastases characteristics in each group (initial and second pulmonary metastasectomy)

Characteristics		The initial pulmonary metastasectomy *n* = 126	The second pulmonary metastasectomy *n* = 26	*p* value
Diagnosis period, *n* (%)		Synchronous 41 (32.5) Metachronous 85 (67.5)	–	–
Number of pulmonary metastasis, *n* (%)		1: 89 (70.6) 2: 17 (13.5) 3: 12 (9.5) 4: 3 (2.4) 5: 2 (1.6)	1: 16 (61.5) 2: 7 (26.9) 3: 1 (3.8) 4: 2 (7.7)	0.36
	Solitary	89 (70.6)	16 (61.5)	0.27
	Multiple	34 (27.0)	10 (38.5)	
Location, *n* (%)	Unilateral	108 (85.7)	23 (88.5)	0.71
	Bilateral	18 (14.3)	3 (11.5)	
Disease free interval (days), median [range]		541.5 [0–4664]	409 [27–1334]	0.13
Maximum tumor size (mm), median [range]		12.0 [5–70]	12.0 [8–40]	1.00
Mediastinal lymph node metastasis, *n* (%)	Negative	117 (92.9)	25 (96.2)	0.95
	Positive	5 (4.0)	1 (3.8)	
Preoperative CEA level, *n* (%)	Normal	86 (68.3)	15 (57.7)	0.61
	Elevated	36 (28.6)	8 (30.8)	
Preoperative CA19-9 level, *n* (%)	Normal	97 (77.0)	21 (80.8)	0.74
	Elevated	12 (9.5)	2 (7.7)	
Chemotherapy before operation, *n* (%)	Yes	28 (22.2)	9 (34.6)	0.09
	No	96 (76.2)	15 (57.7)	
Chemotherapy after operation, *n* (%)	Yes	40 (31.7)	9 (34.6)	0.50
	No	74 (58.7)	12 (46.2)	
Recurrence after pulmonary metastasectomy, *n* (%)	Yes	67 (53.2)	17 (65.4)	0.07
	No	59 (46.8)	7 (26.9)	
Recurrent organ, *n* (%)		Lung 47 (37.3) Liver 12 (9.5) Abdominal lymph node 6 (4.8) Pelvic local recurrence 6 (4.8) Bone 5 (4.0) Brain 5 (4.0) Peritoneal dissemination 3 (2.4) Pleural dissemination 2 (1.6) Bone marrow 1 (0.8) Adrenal 1 (0.8)	Lung 13 (46.4) Pleural dissemination 2 (7.2) Thoracic lymph node 2 (7.2) Liver 1 (3.6) Bone 1 (3.6) Brain 1 (3.6) Pancreas 1 (3.6)	

*CEA* carcinoembryonic antigen level (normal upper limit at 5 ng/ml), *CA19-9* carbohydrate antigen 19-9 level (normal upper limit at 37 ng/ml)

^*^p < 0.05; ^**^p < 0.01; ^***^p < 0.001

### Surgical characteristics in initial and second pulmonary metastasectomy

Surgical characteristics for each group are presented in Table 3. The amount of intraoperative blood loss was significantly higher in patients undergoing second pulmonary metastasectomy {median 10 (range 0–1130) ml vs 20 (0–220) ml, *p* = 0.008}. However, there were no significant differences in postoperative complications, postoperative mortality, or hospital stay between the groups.

**Table 3:** Surgical characteristics in each group (initial and second pulmonary metastasectomy)

Characteristics		The initial pulmonary metastasectomy (*n* = 126)	The second pulmonary metastasectomy (*n* = 26)	*p* value
Operation, *n* (%)	Partial resection	65 (51.6)	8 (30.8)	0.09
	Segmentectomy	31 (24.6)	11 (42.3)	
	Lobectomy	30 (23.8)	5 (19.2)	
	VATS	106 (84.1)	17 (65.4)	0.12
	Open	20 (15.9)	7 (26.9)	
Operation time (min), median [range]		163 [40–645]	217 [62–505]	0.15
Intraoperative blood loss (ml), median [range]		10 [0–1130]	20 [0–220]	0.008^**^
Preoperative %VC (%), median [range]		109.8 [56.7–154]	109.0 [56.4–147]	0.23
Preoperative FEV_1.0_% (%), median [range]		74.9 [46.8–102.6]	72.7 [59.8–97.1]	0.99
Preoperative respiratory dysfunction, *n* (%)	Absent	77 (61.1)	13 (50.0)	0.91
	Present	44 (34.9)	10 (38.5)	
	Restrictive	4 (3.2)	2 (7.7)	
	Obstructive	39 (31.0)	8 (30.8)	
	Mixed	1 (0.8)	0 (0.0)	
Postoperative complication (≧ CD^a^-grade2), *n* (%)	Yes	5 (3.9)	0 (0.0)	0.32
	No	115 (91.3)	24 (92.3)	
		Fistula 2 (1.6) Pneumonia 1 (0.8) Air leakage 1 (0.8) Pleural effusion 1 (0.8)	–	
Postoperative mortality, *n* (%)		0 (0.0)	0 (0.0)	–
Hospital stay (day), median [range]		7 [2–55]	8 [3–19]	0.51

*VATS* Video-assisted thoracic surgery, *%VC* percent vital capacity, *FEV*_*1.0*_*%* forced expiratory volume percent in 1 s

^a^ Clavien-Dindo classification

^*^p < 0.05; ^**^p < 0.01; ^***^p < 0.001

### Survival following the pulmonary metastasectomy from CRC

The median follow-up period after the primary pulmonary metastasectomy was 37 months (range 1–209 months). Of the 126 patients, 33 (24.3%) died after pulmonary metastasectomy: 24 (19.0%) of CRC, 8 patients (6.3%) of another disease, and 1 (0.8%) of unknown causes.

**Fig. 2:**
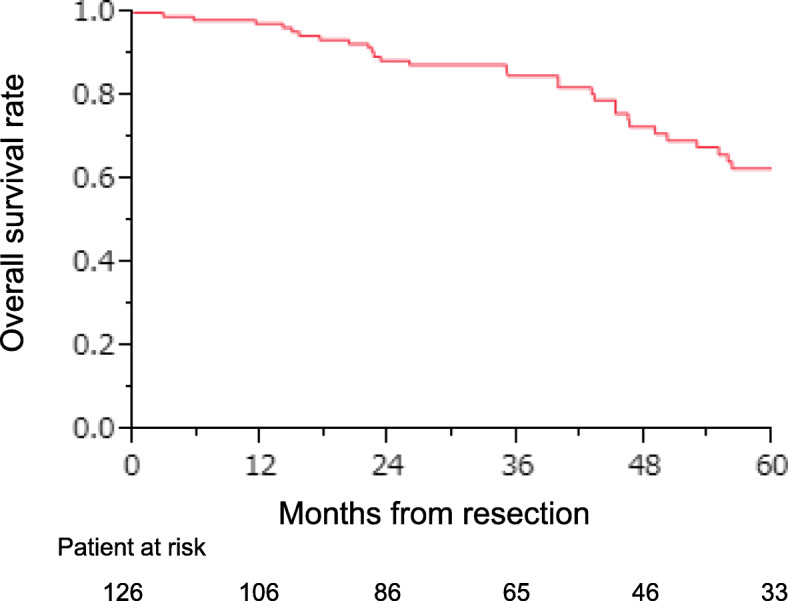
Overall survival of pulmonary metastasectomy for colorectal cancer

**Fig. 3:**
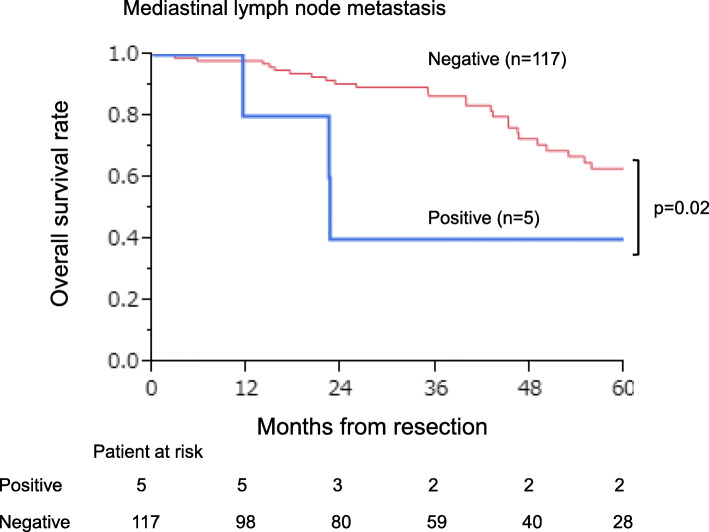
Positive versus negative of mediastinal lymph node metastasis and survival

**Fig. 4:**
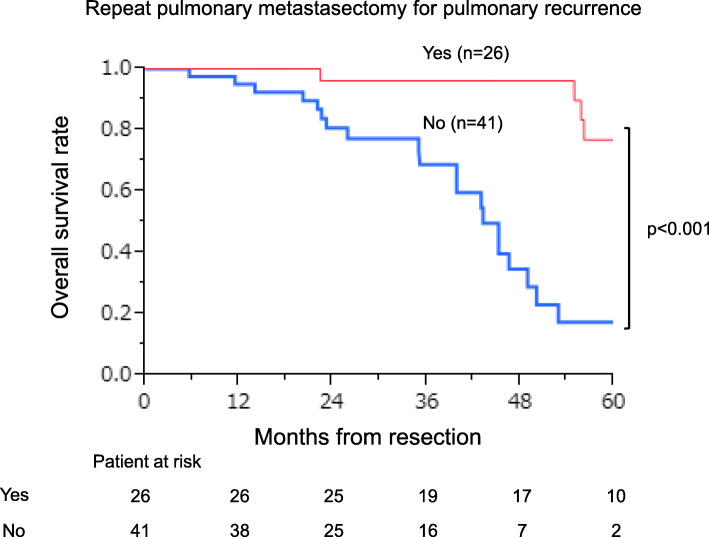
Repeat pulmonary metastasectomy for the pulmonary recurrence and survival.

The 3- and 5-year survival rates of all 126 patients who underwent complete pulmonary metastasectomy were 84.9% and 60.8%, respectively (Fig. 2). Table 4 lists the 5-year survival rates after the pulmonary metastasectomy according to 22 clinicopathological features. Univariate analysis identified seven significant factors: (1) gender (*p* = 0.04), (2) past history of extra-thoracic metastasis (*p* = 0.04), (3) maximum tumor size (*p* = 0.002), (4) mediastinal lymph node metastasis (*p* = 0.02), (5) preoperative CEA level (*p* = 0.01), (6) preoperative CA19-9 level (*p* = 0.03), and (7) repeat pulmonary metastasectomy for pulmonary recurrence (*p* < 0.001). Multivariate analysis using a Cox relative risk regression model indicated that of these features (Table 5), preoperative CA19-9 level was excluded to avoid confounding with preoperative CEA level, and only mediastinal lymph node metastasis (*p* = 0.02, risk ratio 8.206, 95% CI 1.566–34.962, Fig. 3) and repeat pulmonary metastasectomy for pulmonary recurrence (*p* < 0.001, risk ratio 0.054, 95% CI 0.010–0.202, Fig. 4) were significant.

**Table 4:** Survival of the pulmonary metastasectomy from CRC in univariate analysis

Prognostic factors		*n* (%)	5-year overall survival after the initial pulmonary metastasectomy (%)	*p* value
Gender	Male	85 (67.5)	54.5	0.04^*^
	Female	41 (32.5)	77.0	
Age (years)	≧ 70	41 (32.5)	53.8	0.34
	< 70	85 (67.5)	63.1	
Brinkman index^a^	≧ 400	58 (46.0)	64.5	0.78
	< 400	48 (38.1)	59.2	
Primary colorectal tumor location	Colon	59 (46.8)	53.4	0.31
	Rectum	66 (52.4)	69.1	
	Right-sided	23 (18.2)	57.3	0.50
	Left-sided	102 (81.0)	61.3	
Histological differentiation of the primary colorectal tumor	Well	55 (43.6)	67.3	0.26
	Moderately and poorly	59 (46.8)	59.8	
Pathological T stage^b^	T4	37 (29.4)	55.2	0.21
	T < 4	79 (62.7)	63.9	
Pathological N stage^b^	N ≧ 1	71 (56.3)	61.6	0.99
	N0	43 (34.1)	60.5	
Past history of extra thoracic metastasis	Presence	45 (35.7)	53.1	0.04^*^
	Absence	81 (64.3)	65.4	
Past history of liver metastasis	Presence	33 (26.2)	52.4	0.05
	Absence	93 (73.8)	64.3	
Adjuvant chemotherapy after primary colorectal resection	Yes	61 (48.4)	68.5	0.07
	No	57 (45.2)	53.9	
Diagnosis period of pulmonary metastases	Synchronous	41 (32.5)	63.7	0.48
	Metachronous	85 (67.5)	59.3	
Number of pulmonary metastases	Solitary	89 (70.6)	62.2	0.75
	Multiple	34 (27.0)	55	
Location of pulmonary metastases	Unilateral	108 (85.7)	62.9	0.56
	Bilateral	18 (14.3)	52.9	
Maximum tumor size (mm)	≧ 20	27 (21.4)	31.2	0.002^**^
	< 20	94 (74.6)	66.1	
Mediastinal lymph node metastasis	Positive	5 (4.0)	20.0	0.02^*^
	Negative	117 (74.6)	60.8	
Preoperative CEA level	Normal	86 (68.3)	67.7	0.01^*^
	Elevated	36 (28.6)	37.3	
Preoperative CA19-9 level	Normal	97 (77)	64.1	0.03^*^
	Elevated	12 (9.5)	31.8	
Disease free interval after primary colorectal resection (years)	≧ 2	81 (64.3)	59.9	0.91
	< 2	45 (35.7)	62.4	
Chemotherapy before pulmonary metastasectomy	Yes	28 (22.2)	49.6	0.17
	No	96 (76.2)	62.6	
Chemotherapy after pulmonary metastasectomy	Yes	40 (31.7)	60.6	0.77
	No	74 (58.7)	59.7	
Repeat pulmonary metastasectomy for pulmonary recurrence	Yes	26 (38.8)	76.9	<0.001^***^
	No	41 (61.2)	8.7	

*CRC* colorectal cancer, *CEA* carcinoembryonic antigen level, normal upper limit at 5 ng/ml , CA19-9 carbohydrate antigen 19-9 level, normal upper limit at 37 ng/ml

^a^Brinkman index = (the number of cigarette smoked per day) × (the number of years of smoking)

^b^UICC TNM classification (the 8th edition)

^*^p < 0.05; ^**^p < 0.01; ^***^p < 0.001

**Table 5:** Survival of the pulmonary metastasectomy from CRC in multivariate analysis

Prognostic factors	*p* value	Risk ratio	95% confidence interval
Gender (male/female)	0.60	1.324	0.484–4.381
Past history of extra thoracic metastasis (presence/absence)	0.67	1.205	0.511–2.922
Preoperative CEA level (elevated/normal)	0.89	1.083	0.356–3.547
Maximum tumor size (≧ 20 mm/< 20 mm)	0.74	1.203	0.401–3.646
Mediastinal lymph node metastasis (positive/negative)	0.02^*^	8.206	1.566–34.962
Repeat pulmonary metastasectomy for the pulmonary recurrence (yes/no)	< 0.001^***^	0.054	0.010–0.202

*CRC* colorectal cancer, *CEA* carcinoembryonic antigen level, normal upper limit at 5 ng/ml

^*^p < 0.05; ^**^p < 0.01; ^***^p < 0.001

## Discussion

The number of new CRC cases has been increasing annually worldwide. In 2002, the number of new diagnoses was estimated to be about 1.02 million globally [12], but in 2018, the number had increased to about 1.8 million [13]. Accordingly, the number of patients with pulmonary metastases from CRC is inevitably increasing. However, the development of multidrug chemotherapy regimens such as FOLFOX and FOLFIRI and the emergence of molecular targeting drugs such as anti-VEGF antibody and anti-EGFR antibodies have dramatically improved CRC outcomes. Treatment strategies for pulmonary metastasis of CRC have received attention for the purpose of further improving prognosis [1–8].

Previously, pulmonary metastasis was considered to be a condition in which cancer spread throughout the body, and aggressive treatment was commonly avoided. However, since Thomfold et al. [14] proposed the principles of surgical treatment for pulmonary metastases, pulmonary metastasectomy has been performed on patients who meet the operative criteria, and the prognosis after treatment is relatively good. The 5-year survival rate after pulmonary resection is reported to be 30–68% [1–8]; a similar result was observed in this study (60.8%). In the multicenter aggregate in the JSCCR project study [8], the 5-year survival rate of lung resection cases was 46.7% and the cumulative 5-year relapse-free survival rate was 33.7%, whereas the 5-year survival rate of non-resected cases was 3.9%. While many previous reports have shown good results in curative resection for lung metastases from CRC, the PULMICC (Pulmonary Metastasectomy in Colorectal Cancer) trial showed contrasting results [15]. This was a 2-armed multicenter randomized trial which compared pulmonary metastasectomy with continued observation for enrolling 65 patients. As a result, a 4-year overall survival was 40% in the control group versus 43% for patients assigned to metastasectomy. Although this trial was not able to demonstrate a survival benefit of metastasectomy, its outcome may be explained by the small sample size and the good prognosis of the control group which contrasts with the widely held assumption that patients with lung metastases have a 5-year overall survival of < 5% [8, 16]. Thus, it is important to continue to accumulate cohort studies or randomized controlled trials of adequate sample size to reveal the true value of pulmonary metastasectomy.

According to some reports [1–8, 17–27], the number of metastases, location of lung metastases, mediastinal lymph node metastasis, CEA before pulmonary metastasectomy, primary colorectal tumor factors (T factor and N factor), and DFI after resection of the primary colorectal tumor were found to be prognostic factors. In this study, history of extra-thoracic metastasis, maximum tumor size, mediastinal lymph node metastasis, and elevated tumor marker level before pulmonary metastasectomy were also identified as poor prognostic factors. Multivariate analysis identified only mediastinal lymph node metastasis as an independent predictor of poor prognosis. Therefore, excluding cases of mediastinal lymph node metastasis, our results suggest that neither the characteristics of the patients and the primary colorectal tumor nor those of the pulmonary metastases affected the prognosis after pulmonary metastasectomy.

Mediastinal lymph node metastasis in patients with pulmonary metastases is considered to reflect the spread of the cancer to the entire body and is therefore likely to be a poor prognostic factor. In our study, although the number of mediastinal lymph node-positive cases was small, all had past histories of extra-thoracic metastasis. Furthermore, distant metastases to other extra-thoracic organs such as the brain, liver, and bone occurred within 1 year after surgery in these patients. Several studies have suggested an association of mediastinal lymph node metastasis with an increased risk of death [17, 19, 20, 23, 26–28], and a meta-analysis [22] showed poor 5-year survival among patients with lymph node metastasis (range, 0 to 33.5%) compared to those without lymph node metastasis (range, 38.7 to 71%). Our results suggest that lymph node dissection for patients with mediastinal lymph node metastasis has low therapeutic efficacy for those with other poor prognostic factors, and preventive systematic thoracic lymph node dissection to prolong prognosis is probably not necessary. Hamaji et al. reported that systematic lymph node dissection was not a significant factor for prolonged survival in the patients who underwent lymph node dissection, although long-term survivors were present [28]. Furthermore, Welter et al. [29] suggested it is more important to offer adjuvant chemotherapy after metastasectomy in cases of nodal metastasis than to perform radical or systematic lymph node dissection in patients with stage IV disease, bearing in mind the risk of recurrence in extra-pulmonary organs. However, it was reported that CT- and PET-based imaging levels have poor sensitivity (only 35%) for detecting mediastinal lymph node metastasis [28]. In this study as well, the sensitivity of preoperative examination for mediastinal lymph node was 60%, despite a good specificity. Therefore, thoracic lymph node dissection may have clinical significance as a diagnostic tool for prognostic purpose and a direction of the decision towards an adjuvant systemic chemotherapy. In our study, perioperative chemotherapy had no significant effect on survival. Prospective studies on the efficacy of perioperative chemotherapy and appropriate indications for it are necessary in the future.

The present study also showed that repeat pulmonary metastasectomy for pulmonary recurrence is likely to be effective. Repeat pulmonary metastasectomy is a well-established procedure with satisfactory survival [1, 2, 4, 5, 17, 18, 21]. We had 26 patients (20.6% of all 126 cases and 55.3% of the 47 pulmonary recurrent cases after initial pulmonary metastasectomy) who underwent repeat pulmonary metastasectomy. They had 1- and 3-year survivals of 90.7% and 84.6%, respectively, after the second pulmonary metastasectomy, which are similar to the outcomes after initial metastasectomy (97.4% and 84.9%, respectively). There was no significant difference between the two groups in either the clinical characteristics of the primary colorectal tumor and the pulmonary metastases, or in the surgical outcomes including postoperative complications, mortality, and length of hospital stay. Only the amount of intraoperative blood loss was significantly higher in the second pulmonary metastasectomy group, probably because of the higher rate of segmentectomy. Furthermore, the difference in the amount of blood loss was so small that it was considered of no clinical significance. Therefore, at least one repeat pulmonary metastasectomy can be performed relatively safely and can be expected to improve the prognosis if strictly complying with operative criteria.

As a secondary analysis, the prognostic factors for the second pulmonary metastasectomy group were also evaluated by the same method. In past reports [21, 30–32], preoperative CEA level, number of pulmonary metastases, mediastinal lymph node metastasis, and DFI were found to affect survival after repeat pulmonary metastasectomy. In this study, it was difficult to draw a firm conclusion due to sample size and no prognostic factors showing significant differences were found in the patients who met the surgical criteria.

Some limitations of this study have to be addressed. First, the major limitation of our study is the single-institution, retrospective design. Second, there was a potential for selection bias, which was compounded by the retrospective design. Inclusion of patients was highly selective, with patients having presumed good performance status and few comorbidities, which might have contributed to the observed long-term survival. In this study, complete resection of lung lesions is one of the important surgical criteria. Therefore, the surgeon’s aggressiveness, experience, and skill probably have an impact. As a result, some features of pulmonary metastases, such as the number of metastases reported in the past as prognostic factors, may not show significant differences. These limitations should be considered when evaluating the results of the present study. It is necessary to carry out a prospective study with an appropriate control group at multiple institutions that have a unified definition of operative indication and treatment strategy. The medical treatment of malignant tumor has entered the era of immunotherapy [33]. Compared with traditional chemotherapy, neoadjuvant and adjuvant immunotherapy may improve the survival rate of patients. Thus, the study of treatment including immunotherapy will be needed in the future.

## Conclusions

Pulmonary metastasectomy may have a potential survival benefit for patients with metastatic CRC. In our retrospective study, the status of mediastinal lymph nodes was a significant independent prognostic factor. Therefore, the presence or absence of mediastinal lymph node metastases must be accurately determined by thoracic lymph node dissection in cases where lymph node metastasis is suspected from preoperative evaluation and intraoperative findings. Also, a careful follow-up after the initial pulmonary metastasectomy is warranted, because at least one repeat pulmonary metastasectomy may improve the prognosis.

## Data Availability

The datasets used during this study are available from the corresponding author on reasonable request.

## Abbreviations

CRC: Colorectal cancer
JSCCR: Japanese Society for Cancer of the Colon and Rectum
CT: Computed tomography scan
PET: Positron emission tomography
CEA: Carcinoembryonic antigen
CA19-9: Carbohydrate antigen 19-9
DFI: Disease-free interval
VATS: Video-assisted thoracic surgery
%VC: Percent vital capacity
FEV_1.0_%: Forced expiratory volume percent in 1 s
CIs: Confidential intervals
